# Cell Size and the Initiation of DNA Replication in Bacteria

**DOI:** 10.1371/journal.pgen.1002549

**Published:** 2012-03-01

**Authors:** Norbert S. Hill, Ryosuke Kadoya, Dhruba K. Chattoraj, Petra Anne Levin

**Affiliations:** 1 Department of Biology, Washington University in St. Louis, St. Louis, Missouri, United States of America; 2 Laboratory of Biochemistry and Molecular Biology, Center for Cancer Research, National Cancer Institute, National Institutes of Health, Bethesda, Maryland, United States of America; Agency for Science, Technology, and Research, Singapore

## Abstract

In eukaryotes, DNA replication is coupled to the cell cycle through the actions of cyclin-dependent kinases and associated factors. In bacteria, the prevailing view, based primarily from work in *Escherichia coli*, is that growth-dependent accumulation of the highly conserved initiator, DnaA, triggers initiation. However, the timing of initiation is unchanged in *Bacillus subtilis* mutants that are ∼30% smaller than wild-type cells, indicating that achievement of a particular cell size is not obligatory for initiation. Prompted by this finding, we re-examined the link between cell size and initiation in both *E. coli* and *B. subtilis*. Although changes in DNA replication have been shown to alter both *E. coli* and *B. subtilis* cell size, the converse (the effect of cell size on DNA replication) has not been explored. Here, we report that the mechanisms responsible for coordinating DNA replication with cell size vary between these two model organisms. In contrast to *B. subtilis*, small *E. coli* mutants delayed replication initiation until they achieved the size at which wild-type cells initiate. Modest increases in DnaA alleviated the delay, supporting the view that growth-dependent accumulation of DnaA is the trigger for replication initiation in *E. coli*. Significantly, although small *E. coli* and *B. subtilis* cells both maintained wild-type concentration of DnaA, only the *E. coli* mutants failed to initiate on time. Thus, rather than the concentration, the total amount of DnaA appears to be more important for initiation timing in *E. coli*. The difference in behavior of the two bacteria appears to lie in the mechanisms that control the activity of DnaA.

## Author Summary

DNA replication must be coordinated with growth and division to ensure the viability of cells and organisms. In bacteria, it is believed that cell growth–dependent accumulation of the initiator of DNA replication, DnaA, to critical levels determines the timing of initiation. This view is based primarily on data from the model bacterium *E. coli*, which initiates replication only upon achieving a particular size. However, recent data from another model organism, *B. subtilis*, where DnaA is also rate limiting for initiation, suggests that changes in cell size may not impact the timing of DNA replication. This finding prompted us to revisit the relationship between cell size and DNA replication in *E. coli*. While previous studies examined perturbations in DNA replication on cell size, we instead determined the consequences of cell size defects on DNA replication. This converse approach led to the conclusion that, irrespective of size, DnaA needs to accumulate to a critical amount to trigger initiation in *E. coli*, as is generally believed to be the case. In contrast, small *B. subtilis* cells could initiate replication with amounts of DnaA ∼30% less than wild type. Thus, while DnaA is rate limiting for initiation in both organisms, the mechanisms controlling its activity may vary in different bacteria.

## Introduction

Chromosome replication is precisely coordinated with cell growth and division to ensure faithful maintenance of the genetic material. In eukaryotes, a host of cell cycle regulators and checkpoints function in concert to ensure that replication is coupled to growth and division [1]. In bacteria, the prevailing view is that the initiation of DNA replication is linked to the growth-dependent accumulation of the ATP-bound form of the highly conserved protein DnaA. In other words, DnaA-ATP accumulates to an amount sufficient for initiation only by the time cells reach a particular size (mass) [2], [3].

The concept of growth-dependent, rather than cell cycle-dependent, control of DNA replication in bacteria has its origin in the seminal physiological studies in *Salmonella typhimurium* by Schaechter, Maaløe, and Kjelgaard, and in *Escherichia coli* by Cooper and Helmstetter. Combining cell size data from *S. typhimurium* [4] with data on the timing of replication initiation in *E. coli* [5], Donachie deduced that the ratio of cell mass to replication origin at initiation (called initiation mass) was constant in cells undergoing one to three mass doublings per hour [2]. Although the faster growing cells were bigger and had more DNA, they maintained the same origin-to-mass ratio as slower growing cells. Based on this finding, Donachie suggested that growth-dependent accumulation of a positive-acting factor triggers initiation when it reaches a critical intracellular level [2].

Subsequent work identified the highly conserved AAA+ ATPase, DnaA, as the factor stimulating initiation [6], [7]. DnaA-ATP mediates the unwinding of an AT-rich stretch of DNA within the origin, facilitating loading of the replication machinery [8], [9]. In *E. coli*, three forms of negative regulation—sequestration, the regulatory inactivation of DnaA (RIDA), and titration—act in concert to control accumulation of DnaA-ATP and limit initiation to once per division cycle [3]. Sequestration takes place immediately following initiation and is mediated by SeqA binding to hemi-methylated GATC sites in *oriC* and in the *dnaA* promoter. Sequestration prevents DnaA from accessing *oriC* and blocks *dnaA* transcription until the sequestered regions are fully methylated [10]. RIDA, which also plays an important role in regulating DnaA activity, functions during elongation and is mediated by interactions between DnaA, the sliding clamp of DNA Polymerase III, and Hda, which accelerates hydrolysis of DnaA-bound ATP [11], [12]. Finally, titration of DnaA by its binding sites distributed throughout the chromosome keeps free DnaA levels low. One locus, *datA*, has an unusually high affinity for DnaA and is thus thought to play a major role in titration [13].

Through the combined effects of sequestration, RIDA, and titration, the ratio of DnaA-ATP to DnaA-ADP fluctuates over the course of the *E. coli* cell cycle, peaking just before initiation and falling rapidly thereafter, although total DnaA concentration remains more or less constant [3]. Maintenance of initiation mass in *E. coli* is thus explained by growth-dependent changes in the ratio of active/inactive DnaA rather than growth-dependent increases in total DnaA. Consistent with this idea, overreplication and a decrease in cell size at initiation are seen in DnaA or Hda mutants defective in DnaA-ATP hydrolysis [11], [14]–[16].

Although DnaA had been implicated as the primary regulator of DNA replication in other organisms, support for a cell mass-dependent initiation control mechanism outside of *E. coli* is limited to *Bacillus subtilis* [17]–[19]. Like *E. coli*, exponentially growing *B. subtilis* maintains a constant origin to cell mass ratio over a range of growth rates [20], and increasing the levels of both DnaA and DnaN, the sliding clamp of DNA Polymerase III, leads to premature initiation and altered cell size [21], [22]. In contrast to *E. coli*, increases in expression of DnaA in the absence of DnaN in *B. subtilis* engenders a host of pleiotropic and deleterious effects including misregulation of *dnaA* and *dnaN* expression and induction of the SOS response [21], [22].

Despite these similarities, the molecular mechanisms governing initiation seem to differ between *B. subtilis* and *E. coli*. *B. subtilis* lacks a *seqA* homolog, and its DnaA is synthesized in a burst following replication initiation [23]. *B. subtilis* also lacks *hda*. YabA, the putative functional homolog of HdaA, does not appear to alter DnaA-mediated ATP hydrolysis or the accumulation of DnaA [24], [25]. Instead, YabA appears to help prevent premature initiation through two related mechanisms: 1) tethering DnaA to the sliding clamp during elongation [26]–[29] and 2) limiting the amount of DnaA bound at *oriC* by preventing cooperative binding [30]. The release of DnaN from the replisome, or overproduction of DnaN, inhibits the interaction between YabA and DnaA, permitting increased association of DnaA with *oriC* thereby triggering initiation. In both cases, association of DnaN with the replication fork is critical for preventing premature initiation, providing at least a partial explanation as to why overexpression of DnaA, in the absence of a concomitant increase in DnaN, is deleterious to *B. subtilis* [21], [22]. Another *B. subtilis* protein, Soj, which does not have a functional homolog in *E. coli*, has also been implicated in the control of replication initiation [31]. Soj directly interacts with DnaA yet how it regulates the initiator activity remains unknown [32]. Finally, *B. subtilis* lacks a high-affinity site analogous to *E. coli datA*, although binding sites for DnaA exist throughout the chromosome [33]. Together, these data suggest that DnaA availability and access to *oriC* are controlled differently in *B. subtilis*.

Data from *B. subtilis* mutants that are smaller in size but wild type for growth also suggest that growth-dependent accumulation of DnaA-ATP is not the trigger for initiation in this organism. If this were the case, initiation should be delayed in small-size cells until sufficient DnaA-ATP is available. However, the timing of initiation relative to the generation time is unaffected in diminutive mutants [34].

The discovery that initiation can take place at a reduced cell size in *B. subtilis*, counter to significant circumstantial evidence linking initiation to achievement of specific size in *E. coli*, prompted us to re-examine the link between cell size and initiation in both *E. coli* and *B. subtilis*. Although there is significant data supporting the idea that changes in DNA replication alter cell size in both *E. coli* and *B. subtilis*, the converse, the effect of cell size on DNA replication, has not been explored in any depth. Here, we took advantage of cell size-defective mutants to 1) determine if reductions in cell size impact the timing of initiation in *E. coli* and 2) evaluate the role of DnaA in coordinating replication initiation with cell size. We report that although total DnaA per cell is reduced approximately 30% in both *E. coli* and *B. subtilis* cell size mutants in proportion to the size reduction, initiation is delayed only in the *E. coli* mutants. Thus, the total amount of DnaA must accumulate to a critical level in *E. coli* to generate sufficient active DnaA to trigger initiation and coordinate replication with the cell cycle. In contrast, since the *B. subtilis* mutant initiated with only 70% of wild type DnaA, it appears that the timing of replication is governed by cell-cycle dependent changes in the availability of DnaA for initiation, rather than the absolute amount of DnaA.

## Results

### Mutants with altered cell size

To clarify the role of cell mass in coordinating initiation with cell growth and division we employed cell-size mutants, two of *E. coli* and one of *B. subtilis*. *E. coli* mutants included one with a loss-of-function mutation in the gene encoding phosphoglucomutase, *pgm*::*kan*, and another with a gain-of-function mutation in the cell division gene *ftsA*, ftsA* (the gift of Bill Margolin). We selected the *pgm*::*kan* and ftsA* alleles on the basis of reports indicating that cell size is reduced by ∼25% [35],[36] (see Table S1 for a description of strains). For *B. subtilis*, we employed a loss-of-function mutation in *pgcA* (*pgcA*::*Tn*10), the homolog of *E. coli pgm*, which we initially characterized as part of a study on the growth rate regulation of cell size [34]. Importantly, although the mechanism by which *pgm* modulates cell size has yet to be determined, both ftsA* and *pgcA* impact division through direct effects on the division machinery in *E. coli* and *B. subtilis*, respectively [34], [37].

**Figure 1:**
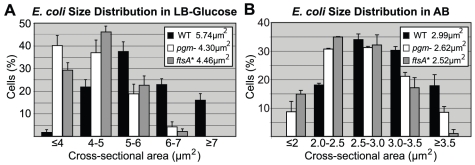
Distribution of cell size. Histograms of wild type and mutant *E. coli* cell size after growth in either (A) LB-glucose or (B) AB. Here, cross-sectional areas were used as proxy for cell size and the mean sizes are shown in the inset. Note that the size distributions of both the *pgm*::*kan* and ftsA* cells are shifted to the left. Experiments were done in triplicate counting >200 cells per strain. Error bars equal one standard deviation. The mutants were significantly smaller in both LB-glucose (p<0.001) and AB (p<0.01) by chi-square analysis.

The size defect of the *E. coli* mutants was most pronounced when cells were cultured in nutrient rich medium (Luria Broth (LB)+0.2% glucose, referred to as LB-glucose). In this medium, *pgm*::*kan* cells were 26±2.7% smaller than wild type and the ftsA* mutants 22±1.3% smaller (Figure 1A; Figure S1A). Consistent with a reduction in cell size, there were ∼20% more mutant cells than the parental strain, when normalized for optical density and assayed by hemocytometer counts (Table 1). In a less rich medium (AB minimal medium+0.2% glucose, referred to as AB), the size reduction was less pronounced but nevertheless significant. In this medium, *pgm*::*kan* and ftsA* mutant cells were 12±2.6% and 17±0.9% smaller than wild type, respectively (Figure 1B; Figure S1B). Growth in a third medium (LB alone) resulted in an intermediate phenotype (data not shown). Both the *pgm*::*kan* and ftsA* strains exhibited mass doubling rates indistinguishable from the MG1655 parent strain in all three media (Figure S1C, S1D), indicating that mutants are normal for growth. In confirmation of our previous work [34], the *B. subtilis pgcA*::*Tn*10 cells were 35±3.3% smaller than wild type cells during growth in LB and 21±1.3% smaller than wild type during growth in a minimal defined medium (S7_50_+1% glucose).

**Table 1:** Initiation age and mass of *E. coli* and *B. subtilis* cells in LB.

	Generation time (min)	Initiation age (0≤age≤1)	Size at initiation (µm^2^)	Cell number per mL (relative mass)^a^
***E. coli***				
Wild type	25.4	0.17	4.49	6.4×10^8^±2.9×10^8^ (1)
*pgm*::*kan*	26.2	0.47	4.19	8.3×10^8^±4.2×10^8^ (0.78)
ftsA*	25.7	0.38	4.29	7.8×10^8^±2.7×10^8^ (0.82)
***B. subtilis***				
Wild type	21.0	0.66	4.68	6.2×10^8^±1.6×10^8^ (1)
*pgcA*::*cat*	21.3	0.61	3.20	8.9×10^8^±2.8×10^8^ (0.69)

a Cell number per OD measured by hemocytometer.

### Reduction in cell size leads to a delay in the onset of DNA replication in *E. coli* but not *B. subtilis*

To determine the timing of DNA replication initiation in *E. coli* and *B. subtilis* mutants, we examined DNA content of cells by flow cytometry after inhibiting replication initiation and cell division with antibiotics. In this method, already initiated replication forks continue and complete replication (replication run-out), yielding fully replicated chromosomes. At the end of the experiment, cells that have initiated replication contain twice as much DNA than cells that have not. In this method, a delay in replication initiation should increase the proportion of uninitiated cells.

**Figure 2:**
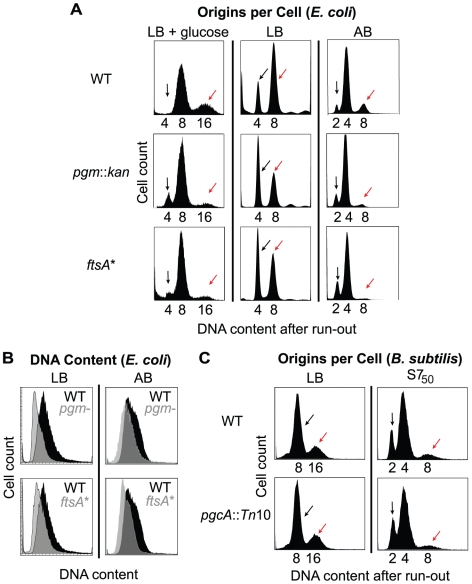
Examination of initiation timing in *E. coli* and *B. subtilis*. (A) Flow cytometry of *E. coli* wild-type and mutant cells grown after replication run-out in either LB+glucose (*left*), LB (*middle*), or AB (*right*). Black arrows indicate the cells that have not initiated replication at the time of drug addition, and red arrows indicate cells that have. Note the increased size of the uninitiated peak and decreased size of the initiated peak in the *pgm*::*kan* and ftsA* mutant cells indicating that initiation is delayed in these strains. (B) Pairwise comparison of total DNA contents of (unsynchronized) log phase cells grown in either LB (*left*) or AB (*right*) in the absence of drugs. DNA content for wild type is in black and grey for both *pgm*::*kan* and ftsA*. (C) Flow cytometry of the *B. subtilis* strains after replication run-out in either LB (*left*) or S7_50_ (*right*). Note the small *B. subtilis* mutant has the wild-type equivalent origins per cell, indicating the timing of replication initiation is normal in this background.

In *E. coli*, both *pgm*::*kan* and ftsA* mutant cells exhibited a distinct delay in replication initiation in all examined conditions (Figure 2A). After replication run-out in LB, both wild type and mutant cells showed two major peaks (Figure 2A, *middle*). The peak on the left (black arrow) corresponds to those cells that have not initiated replication whereas the peak on the right (red arrow) corresponds to the cells that have. Significantly, the peak representing cells that have not initiated DNA replication was larger in both the *pgm*::*kan* and ftsA* mutant populations relative to the same peak in a wild type population in all three media examined. Calculations of initiation age (see Materials and Methods) indicate that in LB, the *pgm*::*kan* and ftsA* mutants delayed initiation by ∼7.9 minutes and ∼5.5 minutes, respectively, relative to wild type cells (when generation time was ∼25 minutes).

Replication run-out of wild-type, *pgm*::*kan* and ftsA* cells cultured at slightly faster (using LB-glucose) or slower (using AB) growth rates also revealed a delay in initiation in the mutant strains (Figure 2A). In LB-glucose, where the size disparity between the wild type and the mutants is maximal, replication run-out revealed three peaks representing cells with 4, 8, or 16 chromosomal equivalents (Figure 2A, *left*). Roughly two-thirds of wild-type *E. coli* cultured under these conditions had 8 chromosomes with the remaining one-third containing 16 chromosomes (mean chromosome number, 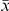, was 10, from three repeat experiments). By contrast, the mutant cells primarily contained 8 chromosomes (*pgm*::*kan*, 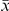 = 8.2 and ftsA*, 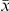 = 8.5). Even in AB, where the size disparity is minimal, there was still strong evidence for an initiation delay in the mutant cells. Under these conditions replication run-out yielded three peaks, representing 2, 4, and 8 chromosome equivalents (Figure 2A, *right*). Relative to wild-type cells, the peak representing 8 chromosomal equivalents (red arrow) was lower and the peak representing 2 chromosomal equivalents (black arrow) was higher in the mutants, again consistent with a delay in replication initiation [(*pgm*::*kan*, 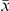 = 4.0 and ftsA*, 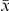 = 3.8) compared to wild type (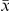 = 4.6)]. The initiation age could not be calculated in these two media because the available algorithms do not apply when the distribution profile has more than two peaks.

Flow cytometry of cells sampled at early-exponential phase but not treated with drugs indicated that the average DNA content of *pgm*::*kan* and ftsA* mutant cells cultured in LB was 72±9% and 81±4% of wild type values, respectively (Figure 2B, *left*). The corresponding values in AB were 87±4% and 82±6%, respectively (Figure 2B, *right*). These results are consistent with an initiation delay in the mutants.

Calculation of cell size at initiation in LB showed that it is more or less equivalent for all three *E. coli* strains (Table 1). These results indicate that *E. coli* needs to achieve an appropriate size for the initiation of DNA replication. In contrast to *E. coli* and in confirmation of our previous observation [34], *B. subtilis* wild-type and *pgcA*::*Tn*10 cells were indistinguishable with regard to the timing of initiation, regardless of growth rate (Figure 2C). Calculations of cell size indicate that the size difference between *pgcA*::*Tn*10 cells and wild-type *B. subtilis* at initiation was the same as at any other point in the division cycle (Table 1). These data support the hypothesis that the cell cycle rather than the cell size governs the initiation of DNA replication in *B. subtilis*.

### DNA replication rates are increased in *E. coli* mutants

Changes in the timing of replication initiation caused by defects in DnaA or SeqA are known to alter C period, the time it takes to complete a round of replication elongation [38], [39]. In particular, earlier initiation leads to an increase in C period, whereas an initiation delay leads to a reduction in C period.

In contrast to SeqA and DnaA, none of the mutations in this study have been directly implicated in the control of DNA replication. However, given the impact of both *pgm*::*kan* and ftsA* on the timing of initiation, we decided to determine the length of C period, as well as D period, the time between the end of replication and cell division, in the small size mutant strains.

We employed marker frequency analysis to determine the length of C and D periods in wild type and mutant cells [40]. C period was derived from quantitative PCR data of the *oriC*-to-*ter* ratio (see Materials and Methods) of early log-phase cells using the following equation:
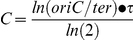
where τ is mass doubling time.

**Figure 3:**
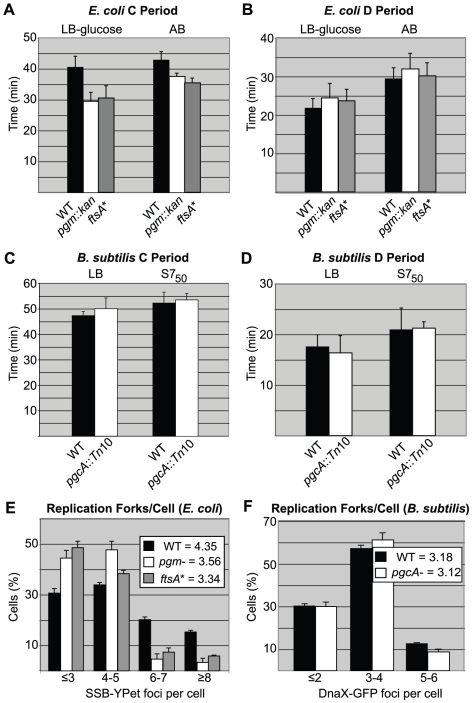
Replication rate is increased in short *E. coli* cells. (A) Average time of DNA replication (C period) is reduced by ∼25% in *pgm*::*kan* and ftsA* cells grown in LB-glucose (*left*) and by ∼15% when grown in AB (*right*). (B) In contrast, C period length is wild type in *B. subtilis pgcA*::*Tn*10 cells in both LB (*left*) and S7_50_ (*right*). The period between the end of replication and division (D period) is wild type in both the *E. coli* and *B. subtilis* mutant strains (C & D). C and D period values were calculated using marker frequency analysis of qPCR data (see Materials and Methods). Error bar corresponds to one standard deviation (n = 3). (E & F) Histograms displaying the number of foci of either single-stranded DNA binding protein fused to YPet (Ssb-YPet) in *E. coli* (E) or a GFP fusion to Tau (DnaX-GFP) in *B. subtilis* (F). Note reduced number of Ssb-YPet foci in *E. coli pgm*::*kan* and ftsA* mutants. Experiments were done in triplicate, assessing 220–260 cells per replicate. Error bars are one standard deviation. Population means are shown in the inset. The *E. coli* mutants had statistically fewer (p<0.001) foci/cell by chi-square analysis.

D period was derived from the following equation using C period from the above equation and origins per cell values as determined by flow cytometry:
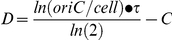



Our calculations indicate that whereas wild-type cells had a ∼40 minute C period in LB-glucose, consistent with previous reports [41], C period was only ∼30 minutes in *pgm*::*kan* and ftsA* mutant cells (Figure 3A). In AB medium, the C period of wild type cells was 42.9 minutes, *pgm*::*kan* 37.6 minutes, and ftsA* 35.5 minutes. The D period remained essentially constant at both growth rates in all cases (Figure 3B).

In contrast to *E. coli*, marker frequency analysis indicated C and D periods are unaltered in the *B. subtilis pgcA* mutant compared to the wild-type cells, both in LB and minimal S7_50_ media, where mass doubling times are ∼22 minutes and ∼37 minutes, respectively. In LB, the C period was ∼50 minutes and D period was ∼17 minutes for both wild-type and *pgcA* mutants (Figure 3C, 3D). In S7_50_ medium, the corresponding values were ∼52 minutes and ∼21 minutes, respectively. Notably, the unchanged cell cycle durations buttress the finding of normal initiation timing for the diminutive *B. subtilis* mutant.

### Origin and replication fork numbers are reduced in *E. coli* mutants but not in the *B. subtilis* mutant

If replication is indeed delayed, as our marker frequency analyses indicate, *pgm*::*kan* and ftsA* cells should have fewer origins and replication forks than congenic wild-type cells. This effect should be particularly noticeable in younger (smaller) cells that have yet to achieve the appropriate initiation mass.

To examine the origin and replication fork frequency in wild type and mutant *E. coli* cells, we employed strains with either a *lacO* array placed near the origin with a cognate LacI-GFP source [42] or a YPet fusion to single-stranded DNA binding protein (Ssb) [43]. Ssb binds to single-stranded DNA just ahead of polymerase to prevent reannealing prior to their replication, and thus can be used as a marker of active replication forks.

In agreement with our C period data, there were fewer replication forks and origin foci in the *E. coli* mutants relative to wild-type cells (Figure 3E; Figure S2A). In rich medium, wild-type *E. coli* had an average of 4.4 Ssb-YPet foci, yet both mutants exhibited on average only 3.5 Ssb-YPet foci per cell. Moreover, while the frequency of cells with six or more Ssb-YPet foci was ∼40% in the wild type population, it was only ∼15% in *pgm*::*kan* and ftsA* mutants. These results suggest a reduction in the number of replication forks in the mutant backgrounds. Similarly, ∼30% of wild type *E. coli* cells had 5 or more origin foci, indicating they had reinitiated replication, but only ∼10% of *pgm*::*kan* cells and ∼3% of ftsA* cells fell into this category. Note that this method underestimates actual origin number relative to replication run-out due to issues associated with chromosome cohesion [44] as well as the limited resolution of conventional light microscopy.

In contrast to *E. coli*, the small *B. subtilis* mutant was wild type both with regard to the number of replication forks and origin foci, consistent with a normal initiation age and normal C period (Figure 3F; Figure S2B). Examining the frequency of foci of GFP-fusion to the replication protein Tau [45], a marker of active replication, we found that ∼30% of both wild-type and *pgcA* mutant *B. subtilis* cells had 2 or less foci, ∼60% have 3 to 4 foci, and ∼10% have 5 or more. Similarly, a *lacO* array at the origin in combination with a cognate LacI-GFP source [46], indicated that both wild type and mutant *B. subtilis* cells have an average of ∼3.2 origins per cell.

### Z period is unaffected by reductions in cell size

To validate our calculations of the cell cycle, we determined the frequency of FtsZ rings in populations of wild-type and mutant *E. coli* strains (see Text S1 for Materials and Methods). FtsZ ring frequency is directly related to Z period, the length of time the cytokinetic ring is present during the division cycle, and the Z period is proportional to D period [47].

Data from strains encoding an inducible FtsZ-GFP fusion indicate that the timing of FtsZ assembly is wild type in *E. coli pgm*::*kan* and ftsA* mutant cells, regardless of growth rate (Figure S2C). Approximately 80% of cells from all three strains (wild type, *pgm*::*kan*, and ftsA*) had FtsZ rings during early-exponential phase in LB-glucose and 70% had FtsZ rings during mid-exponential phase in AB. These results support our finding that D period is normal in the mutant strains. We determined earlier that Z period was wild type in the *B. subtilis pgcA* mutant in agreement with the D period data for this mutant [34].

### Synthesis of DnaA is normal in mutant *E. coli* and *B. subtilis* cells

A simple explanation for the altered initiation patterns of the *E. coli* and *B. subtilis* mutants would be changes in the levels of the initiator protein DnaA. A reduction of DnaA levels in the *E. coli pgm*::*kan* and ftsA* mutants would be consistent with a delay in DNA replication initiation. Conversely, increased levels of DnaA in the *B. subtilis pgcA* mutant would permit them to initiate DNA replication at the same time as their wild-type counterparts, albeit at a reduced size.

**Figure 4:**
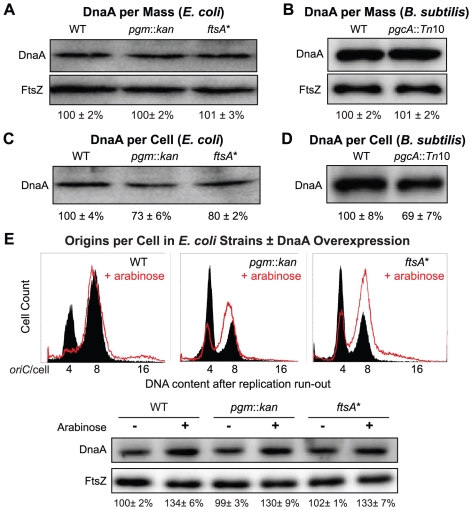
Fewer DnaA molecules in small *E. coli* and *B. subtilis* mutants. (A & B) Quantitative immunoblot of DnaA levels in *E. coli* and *B. subtilis* grown in LB. Gel loading was normalized to optical density. FtsZ serves as loading control. (C & D) Relative DnaA levels per cell. Gel loading was normalized to cell number (see Materials and Methods). Note that while DnaA concentration is wild type in the mutant strains (A & B), DnaA per cell is lower in the mutants (C & D). Error equals one standard deviation (n = 3). (E) Representative plots of DNA content post replication run-out of the *E. coli* strains with a plasmid encoding an arabinose inducible allele of *dnaA* either uninduced (solid black) or induced (red line). Relative concentrations of DnaA are shown at the bottom by immunoblot. FtsZ was used as a loading control.

To address this issue we used quantitative immunoblotting to measure DnaA levels. Gel loading was normalized either to culture optical density to determine the relative concentration of DnaA, or to cell number to determine the relative levels of DnaA per cell. We find that while the concentration of DnaA is wild type in both *E. coli* and *B. subtilis* mutants (Figure 4A, 4B), the calculated DnaA per cell is 30% less, presumably due to their reduced size (Figure 4C, 4D).

These data support the idea that reduced levels of DnaA are the cause of the initiation delay we observed in the *E. coli pgm*::*kan* and ftsA* mutants. On the other hand, the *B. subtilis pgcA* mutant initiated replication on time, despite a reduction in DnaA per replication origin equivalent to the small *E. coli* mutants. This result was somewhat surprising in light of data indicating that artificially altering DnaA levels effects both the timing of initiation and cell size in *B. subtilis* [21], [22]. However, this finding strongly suggests that under normal growth conditions it is the availability of active DnaA rather than its absolute amount that is limiting for initiation in *B. subtilis*.

### Increasing DnaA levels by a modest amount restores normal replication timing in *E. coli*

If reduction in DnaA were the principal cause of replication delay in the small *E. coli* mutants, a modest oversupply of DnaA should restore normal replication timing in the mutants. DnaA was supplied from a low-copy plasmid, pDS596, encoding an inducible allele of *dnaA* [48]. The chromosome number of *pgm*::*kan* and ftsA* cells (red line) increased from an average of ∼5.6 to ∼7 and their initiation age reduced from ∼0.5 to ∼0.2, values congruent to congenic wild-type cells (solid black) (Figure 4E). Similarly, estimates of DnaA molecules per *oriC* between wild-type cells and the mutants overproducing DnaA were nearly equivalent, further supporting the idea that the initiation delay in the mutant cells was due to reduction in DnaA level (Table 2). C and D periods were wild type in mutant cells overproducing DnaA (Table 2), suggesting the increase in elongation rates observed earlier in the mutant strains was a direct consequence of the initiation delay.

**Table 2:** Cell cycle parameters of *E. coli* strains overexpressing DnaA corresponding to Figure 4E.

	*oriC*/*ter*	*oriC*/cell	C period (min)	D period (min)	Initiation age (0≤age≤1)	DnaA/*oriC* b
**WT+** ***P_BAD_-dnaA***						
− arabinose	3.54±0.2	7.0±0.3	43.8±1.2	23.7±1.1	0.25±0.08	196
+ arabinose	4.37±0.3	8.4±0.5	51.1±2.6	22.6±1.3	0.08±0.09	220
***pgm*** **::** ***kan*** **+** ***P_BAD_-dnaA***						
− arabinose	2.59±0.3	5.5±0.3	34.3±4.0	27.2±0.3	0.54±0.02	183
+ arabinose	3.35±0.4	6.9±0.6	43.6±4.6	26.3±0.9	0.20±0.08	197
ftsA* **+** ***P_BAD_-dnaA***						
− arabinose	2.73±0.1	5.76±0.1	34.8±1.9	25.9±1.7	0.47±0.04	187
+ arabinose	3.45±0.4	6.96±0.3	42.8±3.8	24.3±2.2	0.20±0.05	200

b Values were calculated from an estimated number of DnaA per cell from [69], [70] adjusted to the quantitative immunoblots (Figure 4E) then divided by experimentally obtained *oriC* per cell of the same samples.

### The DnaA to *oriC* ratio remains constant with increases in cell size

**Figure 5:**
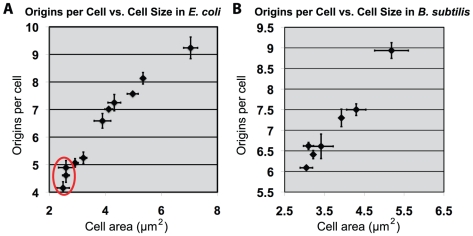
Increasing cell size results in proportional increase of initiations for both *E. coli* and *B. subtilis*. (A & B) Scatter plots of cell size versus replication origins. (A) An *E. coli* or (B) *B. subtilis* strain either overexpressing or depleting the essential division protein FtsZ (see Figure S3 for FtsZ expression levels). Cell origin numbers were determined by examining DNA content post replication run-out by flow cytometry. The median of the population (counts >200) was used for size. Error equals one standard deviation (n = 3). Red circle in “A” denotes *E. coli* cells that are smaller than wild type due to increased levels of FtsZ.

In light of our observation that the small *E. coli* mutants delayed replication due to a reduction in DnaA levels, we were interested to know whether the converse is true: whether DNA replication would initiate earlier in larger *E. coli* cells because they would reach the required initiation size earlier in the cell cycle. Also, how would an increase in cell size impact initiation in *B. subtilis*? To address these questions, we increased cell size by taking advantage of inducible-repressible promoter constructs that permit depletion of the essential cell division protein FtsZ [36], [49]. By titrating levels of inducer (sialic acid for the *E. coli* construct or IPTG for the *B. subtilis* construct) under steady-state conditions we generated cells ranging in size from near wild type to two-fold larger than wild type (Figure 5; Figure S3A, S3B; Text S1). We were also able to generate slightly smaller (15–20%) *E. coli* cells by overexpressing FtsZ ∼60%. However, the same approach was not effective in *B. subtilis*, most likely due to complications from aberrant polar septation events. We also examined the timing of replication in a strain of *B. subtilis* harboring a mutation in the cell division gene *ezrA* (EzrAR510D) that is ∼25% longer than wild type cells but does not have an increase in FtsZ levels [50] (Figure S3C). We examined the timing of initiation of these larger cells by replication run-out and flow cytometry.

Replication initiation earlier in the cell cycle is expected to increase origin number per cell and this expectation was realized (Figure 5). At sizes larger than wild type, replication initiation (*oriC* number) was proportional to cell size. Since DnaA concentration is constant, total DnaA is thus proportional to cell size. These results support the view that as cell size increases, it is the total amount of DnaA that dictates *oriC* number in both *E. coli* and *B. subtilis*. While this result was expected for *E. coli*, it was unexpected for *B. subtilis*. The cell mass independent initiation timing of *B. subtilis* thus could be limited to small cells only. Nevertheless, the identity of the results in the two bacteria when cell sizes exceed the normal range reveals a remarkable property of the regulatory systems to adjust origin contents in proportion to cell mass.

## Discussion

Our investigation evaluating DNA replication initiation in small size mutants of *E. coli* and *B. subtilis* suggests that the mechanisms controlling the timing of initiation differ in these two model organisms. Notably, although total DnaA per cell is reduced by about 30% in the mutants of both strains, only the mutant *E. coli* cells exhibited a delay in initiation. Consistent with growth-dependent accumulation of DnaA-ATP to a critical level being the primary factor governing initiation in *E. coli*, mutant cells delayed initiation until they reached a size more or less equivalent to wild type at initiation (Figure 2A). A modest supply of extra DnaA alleviated the delay, presumably by increasing the levels of DnaA-ATP available for initiation (Figure 4E). In contrast, the *B. subtilis* mutants maintained normal initiation timing without requiring extra DnaA supply (Figure 2C).

### Different approaches to the same problem

Both *E. coli* and *B. subtilis* must coordinate DNA replication with cell growth and division to ensure the production of viable daughter cells. The difference in the initiation timing of the small *E. coli* and *B. subtilis* mutants can be explained by the mechanisms that control the activity and availability of DnaA. In *E. coli*, the inactivation of DnaA-ATP following initiation via RIDA, titration of DnaA by chromosomal binding sites, and the inhibition of *dnaA* expression through sequestration of *oriC*, make growth-dependent accumulation of active DnaA to critical levels the primary trigger for replication [3]. In contrast, our finding that replication timing is maintained in *B. subtilis* despite a 30% reduction in total DnaA, together with mounting evidence that the suite of DnaA regulatory factors are not conserved between *B. subtilis* and *E. coli*, suggests that *B. subtilis* controls initiation primarily by inhibiting DnaA access to *oriC* during elongation. *B. subtilis* is not known to generate DnaA-ADP actively, and DnaA-ATP is most likely the major form of DnaA during the entire cell cycle [51]. Instead, interactions between DnaA and DnaN, YabA, Soj and the primosome proteins DnaB and DnaD, as well as DnaA binding sites on the chromosome appear to be critical for inhibiting DnaA binding to *oriC* and coupling initiation with the cell cycle [24]–[29], [31], [32], [52].

The difference in initiation timing in small cells of the two bacteria can thus be understood if total DnaA concentration can be equated with active DnaA-ATP concentration in *B. subtilis* but not in *E. coli*, as the mechanistic studies in the two systems suggest. Importantly, both mechanisms are sensitive to changes in *dnaA* expression that significantly increase the concentration of active DnaA. In *E. coli*, overexpressing *dnaA* leads to premature initiation by uncoupling synthesis of active DnaA from the cell cycle. In *B. subtilis*, overexpressing *dnaA* presumably leads to saturation of DnaA binding sites on the chromosome and overcomes the activity of inhibitors resulting in premature initiation.

### Rate of replication increases small mutants of *E. coli*

We find that the C period in mutant *E. coli* cells is reduced by ∼25% (Figure 4A, 4E). The causal relationship between the delayed initiation and the reduced C period is not obvious. It is possible that the mutations in the genes such as *pgm* and *ftsA* affect the C period directly, rather than the cell size. If the generation time and the D period remain unchanged and the C period is shortened, then replication ought to initiate later in the cell cycle. This can be viewed using the simulation of the Cooper-Helmstetter model described in the site http://simon.bio.uva.nl/Object-Image/CellCycle/index.html [53]. The opposite view is that the mutations affect the cell size directly, which necessitates delayed initiation to give time for DnaA-ATP accumulation to an appropriate level, and the delay causes the reduction of C period. This latter view is more likely for the following reasons: 1) a similar increase in replication rate was also seen in conditional mutants of DnaA, which initiated replication with a delay [38], [54]. By the same token, premature triggering of initiation by artificially increasing DnaA levels or deleting *seqA* increased the C period [55]. 2) There is as yet no observation that directly links *pgm* and *ftsA* genes to the replication elongation process. On the other hand, there is extensive data indicating that both the *pgm* and ftsA* mutations reduce cell size through direct effects on the cell division machinery in *E. coli* [36]. 3) The orthologous genes, *pgcA* and *pgm*, which both control cell size affected the elongation period only in *E. coli* but not *B. subtilis*. In other words, if the genes were controlling replication, the function has not been conserved. 4) Since the results were same with both *pgm* and ftsA* mutants, it is unlikely that two very different proteins are affecting C period directly and similarly. It should be noted that the Cooper-Helmstetter model does not require that a change in initiation timing entail a change in the C or D period. Since the C period nonetheless does change indicates that there is a homeostatic mechanism in *E. coli* that adjusts the C period in response to the alteration of the initiation time, irrespective of the mechanism by which the initiation time is altered [38], [54].

Mechanistically, how the replication rate can be increased by ∼25% is unclear. The time it takes to replicate the bacterial genome is more or less constant in wild-type cells cultured having doubling times less than 60 minutes, suggesting that elongation is already at maximal speed [5], [56]. We considered the possibility that cells are sacrificing fidelity for speed. However, mutation rates were wild type in both *E. coli* mutants, suggesting fidelity was not compromised (Table S3).

An alternate possibility is that the pool of dNTPs and other replication determinants available for DNA synthesis likely accrue to higher than regular levels when initiation is delayed in *E. coli* [57]–[59]. Since C period depends upon both the fork movement rate as well the time required to restart stalled replication forks, one or both of these processes may have been accelerated as a consequence of initiation delay. The shortening of the C period is unlikely due to an increase in ribonucleotide reductase activity, as *nrdAB* expression is wild type in *pgm*::*kan* and ftsA* E. coli cells (Figure S4; Text S1). Regardless of mechanism, the ability to balance changes in the length of one cell-cycle parameter by altering the length of another is a testament to the inherently homeostatic nature of the replication cycle.

Our data also provide an explanation for a somewhat puzzling result obtained by the Cozzarelli lab [60]. Briefly, Hardy and Cozzarelli identified a loss-of-function mutation in *pgm* in a genetic screen for mutations that reduced negative supercoiling in *E. coli*. Further characterization suggested that the effect of the *pgm* mutant on supercoiling was indirect and thus unlikely to be mediated by Pgm binding to DNA. The potential for an increased-rate replication-fork progression in the *pgm* mutants provides a possible explanation for the observed reduction in negative supercoiling in this strain background. Replication fork progression induces the formation of positive supercoils ahead of the fork [61]. Under normal circumstances topoisomerases reduce this positive supercoiling. We speculate that the increased rate of replication in the *pgm* mutant overwhelms the actions of these topoisomerases leading to an increase in positive supercoiling and a consequent decrease in negative supercoiling. In contrast, *B. subtilis pgcA* mutants have wild-type replication rates (Figure 3C) and exhibit normal levels of negative supercoiling [34].

In contrast to the small mutants, in larger FtsZ-depleted cells from both species the number of origins increased proportionally with cell size (Figure 5). This finding is consistent with the concentration of DnaA remaining constant while the total number of DnaA molecules increases in a manner proportional to cell size. Based on this finding, we propose that the increase in total DnaA per cell following partial FtsZ depletion transiently increases the DnaA/origin ratio, leading to an increase in origin firing and an increase in the number of origins/cell. However, once the DnaA/origin ratio approaches wild-type levels, the new ‘ploidy’ is maintained under steady-state conditions thereby explaining the rightward shift in the replication run-out profile. These results are entirely consistent with the constant initiation mass per origin proposal of Donachie, where growth-dependent DnaA accumulation is the trigger for replication initiation [2]. Importantly, these findings are also consistent with the growing body of data indicating initiation is controlled by cell cycle-dependent changes in the ability of DnaA to access to *oriC* in *B. subtilis* [26]–[29]. In this case, the initial increase in DnaA levels following FtsZ depletion would transiently overcome the ability of inhibitors such as YabA and Soj to prevent DnaA from accessing *oriC* and initiating replication. In any event, the ability to adjust DNA concentration to the cell size appears to be a fundamental property of bacteria, as it is a requirement for stable genome maintenance.

### Implications

Taking advantage of small size mutants of *E. coli* and *B. subtilis*, we demonstrate here that the mechanisms responsible for coordinating DNA replication with cell growth are not conserved in bacteria. The relationship among cell size, DnaA levels, and the initiation of DNA replication has been subject to repeated analysis in *E. coli* and *B. subtilis* and remains to be fully understood (e.g. [21], [22], [39]). In contrast to previous work that principally investigated the effect of perturbations in DNA replication on cell size, ours is the first study to address the converse: the effect of perturbations in cell size on replication. Using this independent and complimentary approach our data reinforces the prevailing view that growth-dependent accumulation of DnaA-ATP is the primary trigger for initiation of replication in *E. coli*. At the same time our approach was essential to reveal that initiation is not tied to a cell size in *B. subtilis*, implying evolutionary divergence in mechanisms of DnaA-dependent regulation, despite DnaA being the regulator of replication in all studied bacteria. Determining the biochemical basis of the contrasting behavior of replication initiation in the two bacteria remains an exciting study for the future. Moreover, given the apparently divergent mechanisms that control initiation in *E. coli* and *B. subtilis*, it will be of great interest to see which strategy is at work in other less studied organisms.

## Materials and Methods

### Strains and media

All *E. coli* and *B. subtilis* strains used are derivatives of MG1655 and JH642, respectively (Table S1). *E. coli* was cultured in Luria Bertani medium (LB), LB+0.2% glucose, or AB minimal medium [62] supplemented with 0.2% glucose, 0.5% casamino acids, 10 µg/ml thiamine, and 5 µg/ml thymidine. *B. subtilis* was cultured in LB or minimal S7_50_ [63] with 1% glucose and the appropriate amino acid supplements. Unless otherwise stated, cultures were started from overnight grown cells, diluted to an OD of 0.005, grown to an OD 0.2–0.6, diluted again to an OD of 0.005, grown to an OD of ∼0.3 for further study.

### Microscopy

This was performed as described [34]. Openlab's density slice module was employed to determine cellular cross-sectional area. Data was corroborated by staining cells with the membrane dye FM4-64 (Invitrogen) at a final concentration of 1 µm/mL and calculating area through length-by-width measurements.

### C and D period determination

*E. coli* or *B. subtilis* cells were grown to an OD_600_ of ∼0.3, treated with sodium azide (300 µg/ml; Fluka BioChemika) and lysed. DNA proximal to the origin (*oriC*) or terminus (*ter*) was amplified by qPCR (see Table S2 for oligonucleotide sequences) and results analyzed using the Pfaffl method [64]. Marker ratios were normalized to the *ori/ter* ratio of either *E. coli* cells treated with rifampicin (300 µg/ml; Sigma) and cephalexin (36 µg/ml; Sigma) or DNA prepared from *B. subtilis* spores [65]. Treatment with chloramphenicol (200 µg/ml; Sigma) for 4+ hours was used for replication run-out in *B. subtilis*.

### Flow cytometry

Flow cytometry was performed as described [66] and origins per cell were calculated using the Cell Quest Pro software and processed in Microsoft Excel. For *B. subtilis*, a *sinI* null or *swrA^+^* revertant background [67] and brief sonication were employed.

### Origins and replication forks per cell

*E. coli* and *B. subtilis* strains either encoding a *lac* operator array near the origin with an inducible source of *lacI*-*gfp*, or carrying a fluorescent protein fused to a replisome component, have been described [42], [43], [45], [46]. Cells were grown to OD_600_ 0.25–0.35, stained with FM4-64, and placed on an agarose pad. Cells were scored for number of distinct foci.

### Cell age and size at initiation

The initiation age (a_i_) was calculated by flow cytometry using the following formula: 
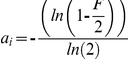
, 0≥*a_i_*≥1; where *F* is equal to the fraction of uninitiated cells [68]. The initiation age was then applied to a distribution of cellular areas to calculate the cell size at initiation. Cell size measured by cross-sectional area correlated well with cell mass measurement by optical density of the culture (Table 1).

### Quantitative immunoblotting

Experiments were performed essentially as described [49]. Briefly, lysates from cultures grown to OD_600_ 0.25–0.35 were normalized to either optical density (600 nm) or cell number (determined using a hemocytometer) and subjected to SDS-PAGE. Immunoblots were performed using either *E. coli* rabbit anti-DnaA antibody (the gift of Jon Kaguni) or *B. subtilis* chicken anti-DnaA antibody (the gift of Alan Grossman), and cognate goat anti-rabbit or donkey anti-chicken secondary antibody conjugated to horseradish peroxidase (Jackson Immunoresearch). DnaA levels were determined relative to FtsZ in individual strains using ImageQuant software and plotted in Microsoft Excel 2008.

### Overexpression of DnaA levels in the *E. coli* mutants

Cells containing pDS596 [48], a low-copy plasmid with an arabinose inducible copy of *dnaA*, were cultured in LB and back-diluted ± arabinose without ampicillin. These strains were evaluated by flow cytometry, marker frequency analysis, and quantitative immunoblotting as described above.

## Supporting Information

Figure S1 Representative pictures and growth curves of *E. coli*. (A & B) Representative fields of wild-type *E. coli* (MG1655) cells and congenic *pgm*::*kan* and ftsA* cells stained with the vital membrane dye FM4-64. Scale bar: 5 µm. Mass doubling of the *E. coli* strains grown in either (C) LB-glucose or (D) AB. Mass doubling time (τ) is in the inset.
(EPS)

Figure S2 Observations of *oriC* and division ring frequencies. (A) *E. coli* and (B) *B. subtilis* strains encoding a *lacO* array positioned near the origin and expressing LacI-GFP. (C) The percentage of the *E. coli* strains grown in LB-glucose (*left*) or AB (*right*) that had a division ring evaluated using a FtsZ-GFP fusion. These experiments were done in triplicate, assessing 220–260 cells per replicate. Error bars are one standard deviation (n = 3). Population means are shown in the inset.
(EPS)

Figure S3 Replication initiation timing in longer cells. (A & B) An immunoblot of FtsZ levels from either (A) *B. subtilis* or (B) *E. coli* with only a xylose or sialic acid inducible copy of FtsZ. Levels of inducer are above, relative expression of FtsZ compared to parental strain are below. DnaA concentrations were indistinguishable at the different FtsZ levels (not shown). (C) Representative replication run-out of wild type, *pgcA*::*cm*, and the EzrA R510D strains. The mutants have the same concentration of DnaA, shown to the left.
(EPS)

Figure S4 mRNA transcript levels of *nrdAB* were measured by qRT-PCR. Error bars equal one standard deviation (n = 3).
(EPS)

Table S1 Bacterial strains and plasmids used in this study.
(DOC)

Table S2 Oligonucleotide sequences used for RT-PCR.
(DOC)

Table S3 Unaltered mutation rate despite faster replication in mutant *E. coli*.
(DOC)

Text S1 Supporting materials and methods.
(DOC)
