# Supplementary material for: Cell Size and the Initiation of DNA Replication in Bacteria
Source: PLoS Genet. 2012 Mar 1;8(3):e1002549. doi: 10.1371/journal.pgen.1002549 (PMC3291569; doi:10.1371/journal.pgen.1002549)
Supplement: Table S1 — Bacterial strains and plasmids used in this study. (DOC) [file pgen.1002549.s005.doc]

**Table S1. Bacterial strains and plasmids used in this study.**

| **Strain** | **Genotype** | **Source or reference** |
| --- | --- | --- |
| ***Escherichia coli* strains** |  |  |
| MG1655 | *F^-^ lambda^-^ ilvG^-^ rfb-50 rph-1* | [1] |
| JW0675-1 | BW25113 *pgm*::*kan* | [2] |
| BH141 | MG1655 *pgm*::*kan* | This work |
| BH317 | BH141 Δ*pgm* | This work |
| WM1659 | TX3772 *ftsA** *leu*::*Tn*10 *tet* | [3] |
| BH142 | MG1655 *ftsA* leu*::*Tn*10 *tet* | This work |
| RRL32 | AB1157 *ssb-ypet kan* | [4] |
| BH321 | MG1655 *ssb-ypet kan* | This work |
| BH318 | BH317 *ssb-ypet kan* | This work |
| BH323 | BH142 *ssb-ypet kan* | This work |
| PB103 (λDR120) | *P_lac_-gfp-ftsZ ap* | [5] |
| BH330 | MG1655 *P_lac_-gfp-ftsZ ap* | This work |
| BH331 | BH141 *P_lac_-gfp-ftsZ ap* | This work |
| BH332 | BH142 *P_lac_-gfp-ftsZ ap* | This work |
| BH397 | MG1655/pDS596 | This work |
| BH398 | BH141/pDS596 | This work |
| BH399 | BH142/pDS596 | This work |
| SG102 | MC1000 *lacO*::*glmS* *kan*/pSG20 | [6] |
| BH413 | MG1655 *lacO::glmS kan*/pSG20 | This work |
| BH415 | BH317 *lacO::glmS kan*/pSG20 | This work |
| BH418 | BH142 *lacO*::*glmS* *kan*/pSG20 | This work |
| PL3180 | W3110 *ftsZ*::*kan*/pWM2765 | [7] |
| BH633 | MG1655 *ftsZ*::*kan*/pWM2765 | This work |
|  |  |  |
| ***Bacillus subtilis* strains** |  |  |
| JH642 | *trpC2 pheA1* | [8] |
| PL1310 | JH642 *pgcA*::*Tn*10 *cat* | [9] |
| BH439 | JH642 *swrA^+^* | This work |
| BH440 | PL1310 *swrA^+^* | This work |
| KPL378 | JH642 *dnaX-gfpmut2 spc* | [10] |
| BH292 | JH642 *dnaX-gfpmut2 spc* | This work |
| BH293 | PL1310 *dnaX-gfpmut2 spc* | This work |
| DCL696 | JH642 *yyaC*::*(lacO cat) thrC*::*(lacI-gfp mls)* | [11] |
| BH301 | JH642 *yyaC*::*(lacO cat) thrC::(lacI-gfp mls)* | This work |
| BH302 | PL1310 *yyaC*::*(lacO cat) thrC*::*(lacI-gfp mls)* | This work |
| PL2084 | *ftsZ*::*spc*, *amyE*::*P_xyl_*-*ftsZ mls* | [12] |
| BH631 | *ezrA*::*ezrA* (R510D) *swrA^+^* | This work |
|  |  |  |
| **Plasmids** |  |  |
| pSG20 | pBAD18 *lacI-gfp* *ap* | [6] |
| pDS596 | pING1 *P_araB_-dnaA^+^ap* | [13] |
| pWM2765 | pACYC184 *P_nahG_-ftsZ ap* | [7] |

1. Guyer MS, Reed RR, Steitz JA, Low KB (1981) Identification of a sex-factor-affinity site in E. coli as gamma delta. Cold Spring Harb Symp Quant Biol 45 Pt 1: 135-140.

2. Baba T, Ara T, Hasegawa M, Takai Y, Okumura Y, et al. (2006) Construction of *Escherichia coli* K-12 in-frame, single-gene knockout mutants: the Keio collection. Mol Syst Biol 2: 2006 0008.

3. Geissler B, Elraheb D, Margolin W (2003) A gain-of-function mutation in *ftsA* bypasses the requirement for the essential cell division gene *zipA* in *Escherichia coli*. Proc Natl Acad Sci U S A 100: 4197-4202.

4. Reyes-Lamothe R, Possoz C, Danilova O, Sherratt DJ (2008) Independent positioning and action of *Escherichia coli* replisomes in live cells. Cell 133: 90-102.

5. Hale CA, de Boer PA (1999) Recruitment of ZipA to the septal ring of *Escherichia coli* is dependent on FtsZ and independent of FtsA. J Bacteriol 181: 167-176.

6. Gordon GS, Sitnikov D, Webb CD, Teleman A, Straight A, et al. (1997) Chromosome and low copy plasmid segregation in *E. coli*: visual evidence for distinct mechanisms. Cell 90: 1-20.

7. Shiomi D, Margolin W (2007) Dimerization or oligomerization of the actin-like FtsA protein enhances the integrity of the cytokinetic Z ring. Mol Microbiol 66: 1396-1415.

8. Perego M, Spiegelman GB, Hoch JA (1988) Structure of the gene for the transition state regulator *abrB*: regulator synthesis is controlled by the *spo0A* sporulation gene in *Bacillus subtilis*. Mol Microbiol 2: 689-699.

9. Weart RB, Lee AH, Chien AC, Haeusser DP, Hill NS, et al. (2007) A metabolic sensor governing cell size in bacteria. Cell 130: 335-347.

10. Lemon KP, Grossman AD (1998) Localization of bacterial DNA polymerase: evidence for a factory model of replication. Science 282: 1516-1519.

11. Lee PS, Lin DC, Moriya S, Grossman AD (2003) Effects of the chromosome partitioning protein Spo0J (ParB) on *oriC* positioning and replication initiation in *Bacillus subtilis*. J Bacteriol 185: 1326-1337.

12. Weart RB, Levin PA (2003) Growth rate-dependent regulation of medial FtsZ ring formation. J Bacteriol 185: 2826-2834.

13. Hwang DS, Kaguni JM (1988) Purification and characterization of the *dnaA46* gene product. J Biol Chem 263: 10625-10632.
