# Supplementary material for: Cell Size and the Initiation of DNA Replication in Bacteria
Source: PLoS Genet. 2012 Mar 1;8(3):e1002549. doi: 10.1371/journal.pgen.1002549 (PMC3291569; doi:10.1371/journal.pgen.1002549)
Supplement: Table S2 — Oligonucleotide sequences used for RT-PCR. (DOC) [file pgen.1002549.s006.doc]

**Table S2. Oligonucleotide sequences used for RT-PCR.**

| **Gene name (organism)** | **Oligonucleotide sequence** |
| --- | --- |
| *gidA* (*B. subtilis*) | 5’ ACAGCTATGGCCGACACTTGAAACC |
|  | 5’ ATAATCCCTTGTCCCGCAGCTTCTTC |
| *dacC* (*B. subtilis*) | 5’ AGGCAATCTGATCGGTGATGATACGTGG |
|  | 5’ GTGCCTGCATCATAGTCTTCGTTTGGAG |
| *glmS* (*E. coli*) | 5’ TTCACCAGATGGGCAATCACTTCGG |
|  | 5’ ACATTGTGGTGGTGCATAACGGCATC |
| *dpc* (*E. coli*) | 5’ ATGGATCTTCCTGCTATACCGCCAC |
|  | 5’ GGCCAACATTTGCGTCCACAGATAAG |
| *nrdA* (*E. coli*) | 5’ TACGTCAGCATCAAAGCGTCGAAAG |
|  | 5’ GCATGATACCCACCAGTTGCAGATAACC |
| *nrdB* (*E. coli*) | 5’ GGTCGAAACCTGGGCGTTCTC |
|  | 5’ TAGCTGGAGATCCCTTCCGCAC |
