# Supplementary material for: Cell Size and the Initiation of DNA Replication in Bacteria
Source: PLoS Genet. 2012 Mar 1;8(3):e1002549. doi: 10.1371/journal.pgen.1002549 (PMC3291569; doi:10.1371/journal.pgen.1002549)
Supplement: Table S3 — Unaltered mutation rate despite faster replication in mutant E. coli. (DOC) [file pgen.1002549.s007.doc]

**Table S3. Unaltered mutation rate despite faster replication in mutant *E. coli*.** Mutation rates were calculated based on rifampicin resistance by the Luria-Delbruck fluctuation test and the Lea-Coulson median estimator (see SI Materials and Methods).

|  | **Wild type** | ***pgm::kan*** | ***ftsA**** |
| --- | --- | --- | --- |
| **Mutation** **rate** | (1.43 ± 0.76) x 10^-8^ | (1.42 ± 0.36) x 10^-8^ | (1.57 ± 0.38) x 10^-8^ |
