# Supplementary material for: Cell Size and the Initiation of DNA Replication in Bacteria
Source: PLoS Genet. 2012 Mar 1;8(3):e1002549. doi: 10.1371/journal.pgen.1002549 (PMC3291569; doi:10.1371/journal.pgen.1002549)
Supplement: Text S1 — Supporting materials and methods. (DOC) [file pgen.1002549.s008.doc]

**Supporting Information Materials and Methods**

**Z period**

A GFP-FtsZ fusion was employed as previously described [1]. Cells were grown in either LB-glucose or AB with 1mM IPTG to exponential phase (OD_600_ 0.3-0.45), and placed on a 1% agarose/PBS pad. Cells were scored as having a robust division ring. In our background strain, growth rate and cellular length were not significantly affected by the induction of FtsZ-GFP fusion (data not shown).

**FtsZ depletion**

*E. coli* or *B. subtilis* strains with only an inducible copy of FtsZ were grown to mid-log in LB + appropriate antibiotic + a concentration of inducer that yielded a wild type level of FtsZ. Cultures were back diluted to an OD_600_ of 0.001 in LB + antibiotic + varying dilutions of inducer. At early mid-log (0.2-0.4) cells were processed for: 1) replication run-out, 2) size measurements, and 3) immunoblot lysates.

**Measuring transcript levels of *nrdAB* by quantitative real-time PCR (qRT-PCR)**

*E. coli* cells harvested for RNA in early-log (an OD_600_ of ~0.25) with [RiboPure™](http://www.ambion.com/catalog/CatNum.php?1924) Kit (Ambion), treated with the Turbo DNA-Free Kit™ (Ambion), and reverse transcribed for 1 hour at 42˚C using the RETROscript® Kit (Ambion). Template was diluted 10-fold and added to iTaq SYBR Green Supermix (Bio-Rad) and ampliﬁed (with primers listed in Table S2) using an Applied Biosystems model 7500 thermocycler. Results were analyzed using the comparative Pfaffl method [2].

**Estimation of mutation rates**

Mutation rates were determined by the modified Luria-Delbruck fluctuation test [3,4]. In short, 1mL of LB broth containing ~5x10^2^ bacterial cells from overnight cultures were inoculated into 30 tubes and incubated overnight. 25 of the 1mL cultures were plated on LB agar with rifampicin (100 mg/mL). The other 5 tubes were plated on LB only agar to determine the total viable count. Both sets were scored for colonies after 30 h. The Lea-Coulson median estimator method [5] was used to calculate mutations per culture. The mutation rate was then calculated by mutations per culture divided by total number of cells per culture.

1. Hale CA, de Boer PA (1999) Recruitment of ZipA to the septal ring of *Escherichia coli* is dependent on FtsZ and independent of FtsA. J Bacteriol 181: 167-176.

2. Pfaffl MW (2001) A new mathematical model for relative quantification in real-time RT-PCR. Nucleic Acids Res 29: e45.

3. Luria SE, Delbruck M (1943) Mutations of Bacteria from Virus Sensitivity to Virus Resistance. Genetics 28: 491-511.

4. Foster PL (2006) Methods for determining spontaneous mutation rates. Methods Enzymol 409: 195-213.

5. Lea, Colulson (1949) The distribution of the numbers of mutants in bacterial populations. J Genetics: 264-285.
